# An easy-to-build and re-usable microfluidic system for live-cell imaging

**DOI:** 10.1186/s12860-018-0158-z

**Published:** 2018-06-20

**Authors:** Julien Babic, Laurent Griscom, Jeremy Cramer, Damien Coudreuse

**Affiliations:** 1 SyntheCell team, Institute of Genetics and Development of Rennes, CNRS UMR 6290, 2 avenue du Pr. Léon Bernard, 35043 Rennes, France; 2 Cherry Biotech, 6 rue Gurvan, 35000 Rennes, France

**Keywords:** Microfluidics, Live-cell imaging, Control of the cellular environment

## Abstract

**Background:**

Real-time monitoring of cellular responses to dynamic changes in their environment or to specific treatments has become central to cell biology. However, when coupled to live-cell imaging, such strategies are difficult to implement with precision and high time resolution, and the simultaneous alteration of multiple parameters is a major challenge. Recently, microfluidics has provided powerful solutions for such analyses, bringing an unprecedented level of control over the conditions and the medium in which cells under microscopic observation are grown. However, such technologies have remained under-exploited, largely as a result of the complexity associated with microfabrication procedures.

**Results:**

In this study, we have developed simple but powerful microfluidic devices dedicated to live-cell imaging. These microsystems take advantage of a robust elastomer that is readily available to researchers and that presents excellent bonding properties, in particular to microscopy-grade glass coverslips. Importantly, the chips are easy-to-build without sophisticated equipment, and they are compatible with the integration of complex, customized fluidic networks as well as with the multiplexing of independent assays on a single device. We show that the chips are re-usable, a significant advantage for the popularization of microfluidics in cell biology. Moreover, we demonstrate that they allow for the dynamic, accurate and simultaneous control of multiple parameters of the cellular environment.

**Conclusions:**

While they do not possess all the features of the microdevices that are built using complex and costly procedures, the simplicity and versatility of the chips that we have developed make them an attractive alternative for a range of applications. The emergence of such devices, which can be fabricated and used by any laboratory, will provide the possibility for a larger number of research teams to take full advantage of these new methods for investigating cell biology.

**Electronic supplementary material:**

The online version of this article (10.1186/s12860-018-0158-z) contains supplementary material, which is available to authorized users.

## Background

Deciphering cell biological processes and understanding the dynamics that underlie fundamental functions in living cells has greatly benefited from the continual development of advanced methods in microscopy. It has now become clear that the investigation of cellular events in a wide range of time scales is integral to our understanding of cell biology, elucidating immediate responses to various stimuli as well as long-term behaviors. However, while complex time-lapse acquisitions based on state-of-the-art imaging procedures have become a standard approach in the life sciences, the real-time observation of cellular responses to changes in the environment or to specific treatments has not progressed at the same pace. Indeed, most of the strategies that are adapted to such experiments still rely on basic perfusion systems that lack versatility and time resolution. Recently, microfluidic technologies coupled with high-resolution microscopy have brought a number of solutions to the limitations that are intrinsic to standard perfusion setups [1]. For instance, they rely on microvolumes [2], improve the time resolution of the assays [3], are compatible with non-adherent cells [4–6], and allow for multiplexing assays in a single experiment [7]. Importantly, taking advantage of the well-established properties of fluid flow within microchannels [8–10], microfluidic systems also provide the capacity to simultaneously alter several parameters of the cellular environment with unprecedented flexibility, accuracy and dynamics [11–13]. Finally, their emergence has been supported by the ongoing development of advanced chip structures, opening new doors of investigation for researchers [2, 14, 15]. From ever more complex fluidic circuits [16–18] to the use of microsystems for assessing the physical properties of living cells [11], the advantages of microfluidics for the life sciences are undisputed, and the design of novel microdevices has become a dedicated area of research.

Nevertheless, the impact of microfluidics and its popularization in biological research laboratories have not been as significant as may have been predicted [19]. One of the major obstacles is the complexity of the fabrication and usage of these devices. Indeed, the building of microfluidic chips adapted to live-cell imaging is a time consuming process that requires costly equipment, dedicated and controlled clean rooms, as well as specific expertise in microfabrication [20–22]. Furthermore, modulating the flow and composition of the growth medium in a chip while monitoring cells by microscopy is challenging. It relies on a clear understanding of specific concepts including the impact of the fluidic resistance at different points in the network and the relationship between flow rate and fluid velocity, which can generate shear stress in the cells. In addition, flows in microchannels are generally laminar, and the mixing of solutions is limited to diffusion: additional microstructures are therefore often required to enhance mixing efficiency [23]. All together, these different aspects of the use of microfluidic systems have hampered their integration in most biology research groups.

A number of commercial systems have recently tried to circumvent these issues, offering high-end and expensive plug-and-play devices compatible with most standard microscopy setups. However, they do not allow any customization of the fluidic networks, a property that is an integral part of what microfluidics can offer to research in the life sciences. More versatile “home-made” systems have been described, including low-cost paper-based devices [24], wax microchips [25] or easy-to-pattern materials [26]. Simpler channel designs and fluid handling methods have also been used for biological assays [27, 28]. However, the fabrication and utilization of these devices are still time-consuming and rely on equipment that is not always available to non-specialized research labs. Furthermore, most of these systems are not re-usable. Implementing microfluidic technologies in a laboratory therefore remains an endeavor that is challenging and costly. Paradoxically, the race toward complexity and ever more powerful microfluidic devices has lost sight of the needs of a majority of research teams. Indeed, while the ideas of lab-on-chips [14, 29, 30] or even organ-on-chips [31, 32] are promising in the long-run, they do not reflect what is currently critical for a large number of investigations in cell biology. It is therefore crucial to establish methods and protocols for the fabrication and use of simple customizable microsystems by non-experts. This is an essential step for fully taking advantage of these revolutionary tools.

As discussed above, one of the major barriers to the integration of microfluidic approaches in biological research laboratories is the complexity of the microfabrication process. In this study, we have addressed this issue and developed a novel microfluidic system dedicated to live-cell imaging that is easy-to-build, customizable and cost-effective, while possessing many of the advantages associated with microfluidics. This device employs a commercially available elastomer in which different types of microfluidic networks can be easily engineered without high-end equipment. We show that the transparent elastomer layer can be bonded to microscopy-grade glass coverslips and re-used several times, a significant advantage for the implementation of these chips in research laboratories. We then provide evidence that it can be coupled to precise controllers, allowing for rapid, accurate and simultaneous control of various parameters of the cellular environment such as the temperature of the sample and the composition of the growth medium. Importantly, we present proof-of-concept experiments that demonstrate the biocompatibility of such microdevices with both yeast and mammalian model systems. Finally, we show that when applying a constant flow of medium through the chips, these systems are well-adapted for the real-time monitoring of cellular responses to dynamic perturbations of their environment. Taken together, our results describe an affordable, easy-to-build and re-usable microfluidic system that will be an asset for research teams in cell biology and contribute to the expansion of microfluidics in the life sciences.

## Results

### A new material for the simple fabrication of complex microfluidic chips

**Fig. 1:**
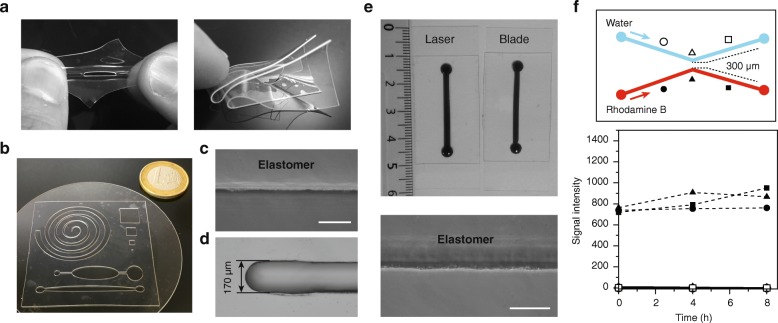
Fabrication of re-usable microfluidic chips using the LSR elastomer. **a.** The LSR elastomer is easy-to-manipulate, resistant to both stretching (left panel) and folding (right panel). **b.** Complex patterns can be fabricated using a CO_2_ laser cutter. A schematic of these patterns is presented in Additional file 4A. **c.** Microscopy image of a laser-cut elastomer sheet showing the precision of the cutting procedure along the edge of the cut. Bright-field image (20×). Scale bar = 100 μm. **d.** The narrowest channel that can be obtained with a 50 μm beam CO_2_ laser cutter is ~ 170 μm wide. A piece of elastomer was placed between 2 liners taken from double-sided adhesive prior to cutting. Bright-field image (10×). Scale bar = 100 μm. **e.** Microchannels can be generated in elastomer sheets using a razor blade and biopsy puncher. Top panel: comparison of similar channels fabricated using a laser (left) and a razor blade (right). A dye is used for ease of visualization. Bottom panel: the use of a razor blade allows for high quality cuts. Bright-field image (20×). Scale bar = 100 μm. **f.** Multichannel microfluidic chips. An elastomer chip comprising 2 channels (500 μm wide) separated by 300 μm at the middle section (left panel) was fabricated and intercalated between two glass coverslips. The two channels were injected with either water or Rhodamine B (5 μM) at a constant flow of 20 μl/min, and the fluorescence intensity at the indicated positions (geometric marks) was measured over time (right panel). *T* = 0 corresponds to 5 min after the start of the perfusion. Data were corrected for the background measured at the entrance of the water channel at T = 0

While a host of complex microdevices and microfabrication procedures have emerged for a broad range of applications [11, 12, 33], the integration and popularization of versatile, custom-made microfluidic systems in research laboratories has been hampered by the prevailing microfabrication techniques and materials, which require high-end equipment, controlled environments and specific expertise [20–22]. We therefore sought to identify an alternative material that would be readily available to researchers and easily integrated in microfluidic chips dedicated to live-cell imaging. Silicone-based materials are particularly adapted for such approaches, as they are robust and their bonding properties allow the sealing of different layers for the assembly of microscopy-compatible microfluidic chips. In order to bypass the requirement for a dedicated clean room as well as advanced fabrication techniques, we focused on materials that are already available as sheets with precise thicknesses, a critical feature for the production of reproducible microfluidic networks. After a pilot screen to assess the basic properties of a number of existing solutions (e.g. bonding characteristics, transparency, short-term biocompatibility; our unpublished observations), the Liquid Silicone Rubber (LSR) elastomer was selected as the most promising candidate. This material can be purchased in a range of thicknesses (from 250 μm to 3.2 mm; all the systems described in this study are 250 μm thick), and it is resistant to stretching and deformation (Fig. 1a), making it particularly suitable for routine manipulation. Importantly, we demonstrated that complex microchannel structures can easily be engineered in thin LSR sheets using a CO_2_ laser cutting machine (Fig. 1b, c) and found that a 50 μm laser beam, which is standard in a number of laser cutters, allows for the generation of channels as narrow as ~ 170 μm (Fig. 1d). Therefore, although very small structures cannot be fabricated, this method is compatible with a broad range of live-cell imaging experiments (as a reference, the size of the sensor chip of wide field sCMOS cameras is 2048 × 2048 pixels, representing ~ 13.3 × 13.3 mm). Undoubtedly, the use of an automated laser cutter provides accuracy and reproducibility at manageable costs. However, such an apparatus is not always available in research laboratories, prompting us to assess the fabrication quality of LSR-based microchips using razor blades and biopsy punchers. Interestingly, we were able to produce simple but perfectly usable channels, whose dimensions are adequate for a majority of standard live-cell imaging assays (Fig. 1e).

Next, we established the compatibility of our strategy with experiments that require the maintenance of medium flow through the microdevice. To this end, we determined the bonding strength of the elastomer when intercalated between two microscopy-grade glass coverslips, using a method that we previously described [13]. Briefly, our assay uses a pressure controller connected to the chip to identify the maximum pressure that can be applied within the microsystem without generating fluid leaks. Importantly, we found that the sealing properties of the elastomer with glass at different physiologically relevant temperatures are sufficient for robust bonding and withstand pressures that are suitable for a broad panel of applications (Tables 1 and 2). Moreover, we obtained identical results between new chips and chips in which the elastomer layer had been re-used several times after thorough washing (see Methods). This showed that beyond its ease of manipulation and fabrication, this material provides re-usability, a clear asset for promoting microfluidic-based approaches in the life sciences.

**Table 1:** Bonding strength of the elastomer

	Temperature (°C)				
Pressure (kPa)	20	30	40	50	60
10	+	+	+	+	+
15	+	+	+	+	+
20	+	+	+	+	+
25	+	+	+	+	+
30	+	+	+	+	+
35	+	+	+	+	+
40	+	+	+	+	+
45	Leak	Leak	Leak	Leak	Leak

Chips as in Additional file 4C were assembled, filled with water and placed on a hot plate at the indicated temperatures. The outputs were then closed, and increasing pressures were applied to the inputs using a high precision pressure controller (10 s at each pressure prior to increase). This allowed for monitoring of the pressure at which the bonding strength of the elastomer is insufficient, resulting in detachment of the material from the glass coverslip and fluid leakage (“leak”). “+” indicates no leak. These experiments were performed using newly-cut chips as well as used chips (> 10 times), and identical results were obtained

**Table 2:** Relationship between the flow rate and the applied pressure

Flow rate (μL/min)	Pressure (kPa)
10	5.6
20	12.9
30	21.4
40	30.3
50	38.8
60	48.0
70	57.0
80	66.7

Chips as Additional file 4C were assembled and filled with water. The flow rates through the chips were then set using high precision flow sensors, and the pressure applied to maintain each flow rate was assessed. This showed that the chip can withstand flow rates of 50 μL/min (see Table 1). Note that the value indicated by the pressure controller in these assays corresponds to that in the flow sensor, which is higher than the pressure in the microsystem itself (as determined in Table 1). The chips are therefore likely to be compatible with flow rates above 50 μL/min

Finally, we investigated the possibility to design elastomer chips that would allow for performing multiple simultaneous assays (e.g. experiment and controls, different treatments of a single cell line). This would involve the fabrication and independent use of distinct channels within a single chip. To test this, we built two channels separated by only 300 μm (Fig. 1f) and intercalated this elastomer layer between two glass coverslips. Water was then injected in one of the channels, while the other was perfused with a solution of the fluorescent dye Rhodamine B. Identical and constant flow rates in both channels were maintained for 8 h (20 μL/min; as a reference, the total volume of liquid in each of these channels is ~ 6 μL), and the fluorescence intensity was measured at different positions (Fig. 1f). Strikingly, we observed no cross-contamination between the channels even at the narrowest point of the network, demonstrating the compatibility of this material with the design and use of multi-channel microsystems.

Taken together, these results demonstrate that the LSR elastomer is a promising candidate for generating microfluidic devices for live-cell imaging, which is compatible with the fabrication of complex channel networks and with experimental multiplexing on single chips.

### Precise and simultaneous control of the environment in the perfusion channel

**Fig. 2:**
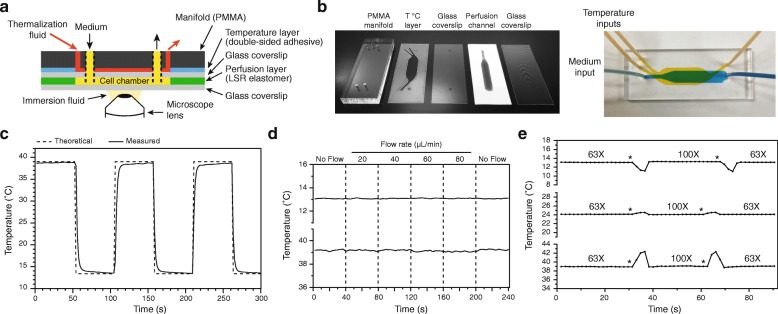
Microfluidic-based temperature control of the elastomer microfluidic chip. **a**. Schematic of an elastomer chip integrating a temperature control. **b.** Left: Layers used to mount the chip in *a*. For the perfusion layer, a white background and a blue dye were used for ease of visualization. Right: assembled chip. An orange dye (temperature control) and a blue dye (perfusion layer) were used for ease of visualization. Connecting tubings were attached to the manifold using epoxy glue. See Additional file 4C for channel dimensions. The temperature control is referred to as T °C layer. **c-e.** All experiments used chips as in Additional file 4B, and all temperature measurements within the cell channels were made using metal electrodes deposited on glass coverslips [13]. **c**. Fast temperature shifts using the temperature control system. A series of switches were triggered (without a constant flow of medium) and the theoretical sample temperature was compared to experimental measurements. The theoretical temperature was calculated based on the calibration equation (Additional file 2) as well as the measured lens and thermalization fluid temperatures. **d.** The temperature of the sample is robust to changes in the medium flow rate. Sample temperature was set below (top) or above (bottom) ambient, and the medium flow rate was altered. No temperature changes were measured, even at the highest flow rate (80 μL/min). **e.** The temperature of the sample is robust to changes in immersion lens. Sample temperature was set below (top), comparable to (middle) or above (bottom) ambient, and the lens was switched back and forth between a 63X and a 100X objective (asterisks: timings of the switches). No medium flow was applied, as it has no impact on the temperature (see *d*). Only transitory changes in temperature were observed, corresponding to the short periods during which the microsystem was not in contact with the objectives

We next investigated whether the rapid, precise and simultaneous control of different parameters of the cellular environment during live-cell imaging experiments can be achieved using our microsystem. To this end, we first generated a proof-of-concept microscopy-compatible chip that integrated 1) a perfusion channel built in a 250 μm-thick elastomer sheet and 2) a microfluidic-based temperature control system that we previously described [13] (Fig. 2a, b). The latter was fabricated in medical double-sided adhesive using the laser cutter, but similarly to the elastomer perfusion layer, it could easily be fabricated using a simple razor blade (Additional file 1). Once calibrated to compensate for the heat losses that occur along the microfluidic network (Additional file 2 and Methods), this additional layer allowed not only the maintenance of a precise and constant sample temperature but also the induction of rapid and reproducible temperature shifts both above and below ambient (Fig. 2c). This is a key feature when studying temperature-sensitive processes or genetic mutants. Importantly, we previously showed that this temperature control system is not significantly influenced by either the flow rate of medium within the cell channel or the type of immersion lens used [13]. However, these experiments were performed with much thinner microfluidic channels and structures than those described in the present study. Nevertheless, we showed that these properties were valid in the elastomer chips as well, for a range of common physiological temperatures (Fig. 2d, e). In fact, we observed that higher perfusion flow rates could be applied in the elastomer microsystems (60 μL/min affected the temperature in our previous devices [13], while the chips presented here could accommodate the maximum tested flow rate of 80 μL/min). Thus, the elastomer microfluidic chips are compatible with precise and dynamic regulation of sample temperature while monitoring cell behavior by microscopy.

**Fig. 3:**
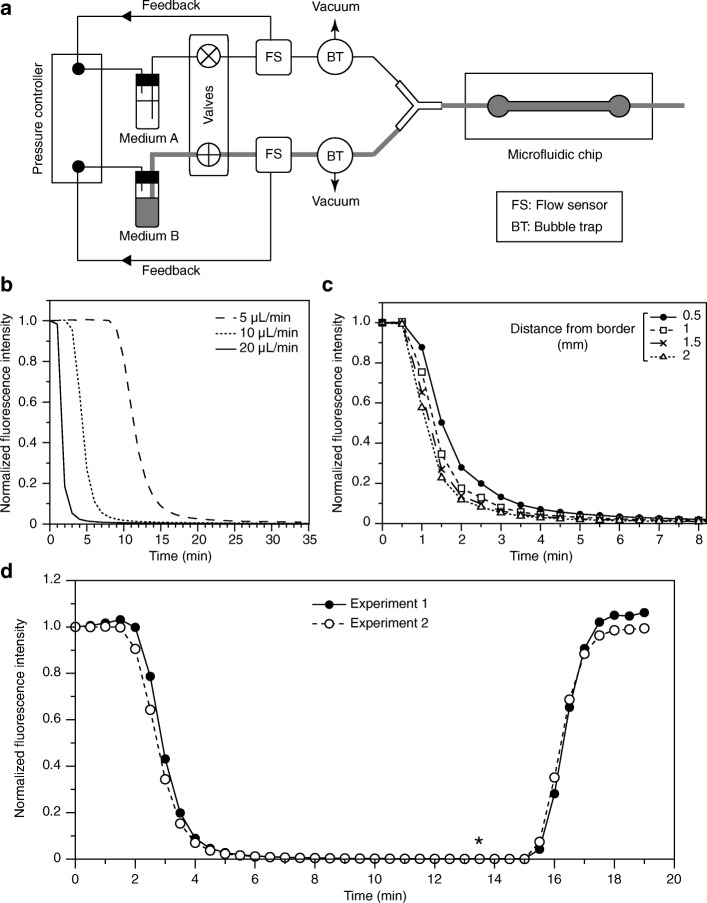
Fast media switches using the elastomer microchips. **a**. Schematic of the setup used for switching media. Flow sensors (FS) modulate the operation of a pressure controller to maintain an accurate flow rate. Media switches are achieved using valves. Vacuum-based bubble traps (BT) limit the presence of bubbles (BTs were added when using cells but not in *b*-*d*). **b-d**. All experiments used chips as in Additional file 4C. **b.** Media switch dynamics. 50 μM Rhodamine B was perfused in the chip (20 μL/min) for 10 min. Water was then injected (20 μL/min, *T* = 0), and fluorescence intensity was followed over time. The chip was then re-used twice with the same protocol but with water perfusion at 10 μL/min followed by 5 μL/min. Data were corrected for the background (measured prior to the initial Rhodamine B injection) and normalized to T = 0 for each flow rate. **c.** Influence of the position within the channel on the media switch dynamics. 5 μM Rhodamine B was injected (20 μL/min) for 10 min prior to switching to water (20 μL/min, T = 0). Fluorescence intensity was then measured over time at various distances from the channel border. Data were corrected for the background (measured prior to the initial Rhodamine B injection) and normalized to T = 0 for each position. **d.** Reproducibility of the media switch dynamics. 50 μM Rhodamine B was injected (20 μL/min) for 10 min. At T = 0 and *T* = 13.5 min (asterisk), the perfusion was switched to water (20 μL/min) and back to Rhodamine B, respectively. Fluorescence intensity was then followed over time (Experiment 1). The same chip was re-used in an identical experiment (Experiment 2). Data were corrected for the background (measured prior to the initial Rhodamine B injection) and normalized to T = 0 for each experiment

We then assessed the capacity of this perfusion system to perform rapid changes in growth medium. Using Rhodamine B as a fluorescent marker, we first investigated the dynamics of the media switches as a function of the flow rate. This parameter was set to 5, 10 or 20 μL/min (within the range determined in Tables 1 and 2) using high precision flow sensors that modulate the operation of pressure controllers and a matrix of valves to induce the changes in perfused fluid (Fig. 3a). After injecting the dye, a rapid switch to water was applied, and fluorescence intensity in the channel was measured over time. As anticipated, higher flow rates allowed for faster renewal of the medium, with a reduction of 95% of the fluorescence signal after only 4 min when using 20 μL/min (Fig. 3b), while the same was achieved after 8 min and 17 min at flow rates of 10 and 5 μL/min, respectively. However, a lower flow velocity within the chip can sometimes be preferred when shear stress in the cells is more critical than the timing of the medium switch. Interestingly, for each condition, when we considered the last point of the plateau in signal intensity and the first point where more than 90% of the fluorescence is lost, we noted timings of 3, 5 and 7 min for flow rates of 20, 10 and 5 μL/min, respectively (Fig. 3b). This suggests that the delays in medium renewal at lower flow rates result in large part from the time required to displace the volumes of fluid in the connecting tubings rather than within the elastomer perfusion channel. Limiting the length of these tubings is therefore likely to reduce the duration of the media switches when using low flow rates.

As border effects and parabolic flow profiles are often observed in microfluidic devices [8, 10], we set out to assess the homogeneity of the media switches along an axis perpendicular to the flow. We performed the same assay as above (flow rate = 20 μL/min) but determined the fluorescence intensity over time at various distances from the edge of the channel (Fig. 3c). This showed that the position within the channel had only a limited influence on the dynamics of medium exchange. The border effect is therefore likely to be negligible for most applications and only a concern when it is essential to have a high level of homogeneity in the channel. Finally, we demonstrated the reproducibility of the media switch dynamics by performing successive renewals within the same microdevice using the same Rhodamine B assay (Experiments 1 and 2 in Fig. 3d). Importantly, although all the experiments presented above were made with a proof-of-concept design, our conclusions are applicable to other networks as well, with only minor additional characterizations and optimizations. Furthermore, simple gravity-based flows and manual valves can easily be used to renew and switch the fluids in the chips, although they do not provide the degree of precision and reproducibility of more high-end controllers.

While the LSR elastomer cannot be compared to demanding photolithography processes, as it does not allow the fabrication of extremely small channels and multilayered structures, our results show that it represents an ideal material for the fabrication of simpler but powerful microsystems. In addition, the possibility to re-use the elastomer layer multiple times is an important advantage, making these microdevices cost-effective, easy to use, and more likely to become integrated in biology laboratories.

### Live-cell imaging using the elastomer chips

All the assays described above demonstrate the different features of the microsystems that can be assembled using the elastomer, from their robustness to the possibility of dynamically controlling a range of environmental parameters. However, the goal of this study was to establish a new and easy-to-fabricate system dedicated to live-cell imaging. We therefore set out to validate the optical properties of the elastomer. Indeed, although cells under microscopic observation in the perfusion channel are only separated from the microscope lens by a microscopy-grade glass coverslip, strong autofluorescence from the material would interfere with experiments using quantitative fluorescence measurements. To test this, we injected medium in a chip, directly exposed the elastomer surrounding the perfusion channel to the most common excitation wavelengths, and compared fluorescence intensities to those measured within the channel. Strikingly, we found no differences in signal between compartments, demonstrating that the optical properties of the elastomer are particularly suited for fluorescence microscopy (Table 3).

**Table 3:** Autofluorescence of the elastomer

Excitation wavelength	Glass	Elastomer
405	100.7 ± 1.8	100.9 ± 1.8
445	102.3 ± 2.2	102.8 ± 2.3
488	104.5 ± 2.7	104.9 ± 2.7
515	101.6 ± 1.9	101.6 ± 2.1
561	101.5 ± 2.0	101.6 ± 2.0

Cells grown in culture medium were injected in a chip (as in Additional file 4C) to determine the appropriate focal distance for fluorescence quantification. The observation area was then moved to the elastomer to measure its autofluorescence. Culture medium between two glass coverslips was used as a control (glass). Averages of the fluorescence over more than 2000 pixels along a diagonal line scan with standard deviations are shown for the indicated excitation wavelengths. Note that potential autofluorescence from the elastomer would only minimally affect imaging, as by design, there is no elastomer in the light path (see Fig. 2a)

**Fig. 4:**
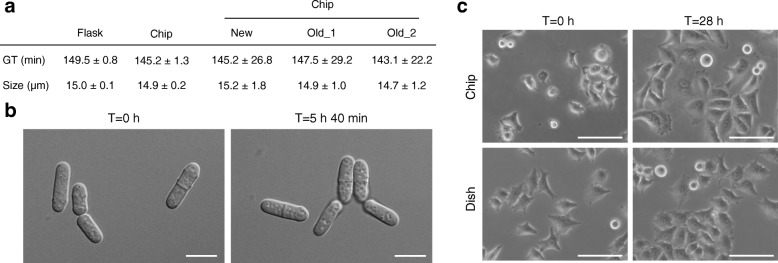
Biocompatibility of the elastomer microfluidic chips. **a**, **b**. All experiments used chips as in Additional file 4C. **a.** Fission yeast cells were injected in a lectin-coated microchip, and medium was perfused (20 μL/min) at 32 °C. After 2 h, images were acquired over > 5 h to calculate generation times and cell sizes at division. Results from a newly cut elastomer chip were compared to those obtained with re-used chips (> 10 times) and in control batch cultures. For each parameter in the first two columns (flask and chip), the average of 3 independent experiments is shown with the standard error. Size at division: *n* ≥ 50. Generation time in chips: *n* > 20. Data for individual chips (last three columns) are shown with their standard deviations. Old: re-used chips. **b.** DIC images of cells grown in the chips in *a* at the indicated times. Scale bars = 10 μm. **c.** HeLa cells were injected in a chip or in a standard culture dish at similar densities and grown for 28 h at 37 °C. A constant flow of medium (5 μL/min) was applied in the chip after cells were allowed to adhere to the glass (~ 3 h after injection, *T* = 0). At T = 0, 5.4 and 8.2% of cells were in mitosis (rounded or in duplets prior to cytokinesis) in flask and in chip, respectively (*n* ≥ 100). At *T* = 28 h, 2.8 and 7.5% of cells were in mitosis in flask and in chip, respectively (*n* ≥ 100). The chip used the perfusion channel as in Additional file 4C but no temperature channel (see Methods): to simplify this proof-of-concept assay, sample temperature was maintained by a high precision hot plate. For this, chip and tubings were pre-incubated overnight with 2 mg/mL BSA at 4 °C and washed with medium prior to cell injection. Bright-field images. Scale bars = 100 μm

Next, we assessed the behavior of living cells grown in our microsystems in order to determine the biocompatibility of the elastomer. First, we injected fission yeast cells (*Schizosaccharomyces pombe*) in a chip after coating the bottom coverslip with lectin to promote cell adhesion to the glass substrate. We previously observed that *S. pombe* cells grown in very confined environments without medium renewal show various phenotypes, including a reduction of their size at division (our unpublished observations). Thus, a constant flow of 20 μL/min of fresh medium was applied and cells were grown in these conditions at 32 °C for several hours. While we surmised that the renewal of medium may circumvent this issue, the shear stress imposed by such a flow may have other deleterious effects on cell physiology. Using this setup, we therefore determined potential alterations in division time as well as changes in cell size at division and in cell morphology. All these phenotypes are well-described markers that allow the identification of defects in cell cycle progression and cell organization [34, 35]. Comparing cells dividing in both new and re-used microfluidic chips with cells grown in standard batch cultures, we observed no differences for any of these properties after more than 5 h (Fig. 4a, b). This showed that the elastomer chips are compatible with the use of fission yeast cells and that the application of a constant flow of fresh medium does not appear to affect cell growth.

Finally, we tested the use of the elastomer chips with adherent mammalian cell lines. To this end, we seeded HeLa cells in either our microsystems or standard cell culture-grade 12-well plates. While the plates were subsequently kept in a controlled CO_2_ incubator without medium renewal, a constant flow of fresh medium (5 μL/min) was applied to the chips after cells had adhered to a fibronectin-coated glass coverslip (~ 3 h after their inoculation). For ease of manipulation in this assay, a temperature of 37 °C was ensured in the microchannels using a high precision hot plate. Cells were then allowed to grow for 28 h in these conditions. As was the case for fission yeast, we did not observe any detectable difference in cell growth between the chips and the plates, with similar increases in cell density, no apparent defects in cell morphology and no increase in cell death (Fig. 4c). Again, not only did this experiment show the compatibility of our devices with the use of mammalian cells, but they also suggested that the shear stress resulting from the maintenance of a constant flow of medium in the chips does not affect these cells.

Collectively, these results demonstrate that our microsystems can be used to perform live-cell imaging experiments with different types of cells without any apparent defects or alterations to their proliferation potential.

### Dynamic modulation of the cellular environment and real-time monitoring of cellular responses

To fully establish our chips as easy-to-build but powerful systems for live-cell imaging experiments, in particular when dynamic modulation of the medium composition is required, we set out to perform different media switches and monitor the responses of cells to these alterations.

**Fig. 5:**
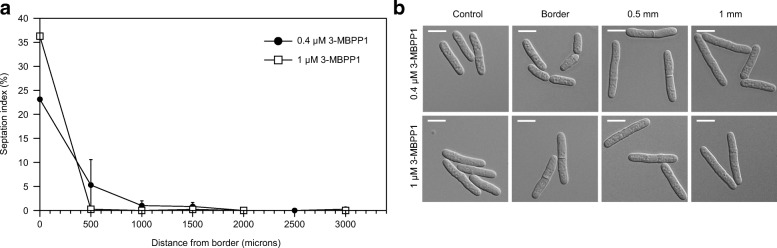
Absorption of small molecules by the elastomer. All experiments were performed using chips as described in Additional file 4C. **a.** The absorption of small molecules by the elastomer restricts the area of the chip that can be monitored when treating cells with such molecules. Fission yeast cells operating with a modified cyclin-dependent kinase (CDK) module that is sensitive to inhibition by the 3-MBPP1 ATP analogue [37] were injected in a lectin-coated microsystem and exposed to a 20 μL/min flow of medium containing 1 or 0.4 μM 3-MBPP1, with sample temperature maintained at 32 °C. After 3 h, septation indexes were determined at different distances from the border of the channel. Graphs represent the averages of 3 independent experiments (*n* > 50 for each experiment) with standard errors. Note that to facilitate this proof-of-concept assay, the sample temperature was maintained using a high precision hot plate. **b.** DIC images of cells in *a* at the border and at distances of 0.5 and 1 mm from the border of the chip. At ≥1 mm from the border of the chip, both concentrations of inhibitor led to a complete G2 arrest, as seen in the control experiments (cells exposed to 3-MBPP1 in standard batch cultures). The size at division of cells at the border when treated with 1 μM 3-MBPP1 was 23.2 μm (average of 3 independent experiments, standard error: 0.7; *n* > 40 for each experiment), which is significantly larger than in inhibitor-free medium (compare with Fig. 4a). This demonstrates that not all of the inhibitor is absorbed by the elastomer. Scale bars = 10 μm

First, we used fission yeast cells operating with an engineered cell cycle control network that is sensitive to inhibition by the ATP analogue 3-MBPP1 [36, 37]. We previously showed that treatment of these cells with high concentrations of 3-MBPP1 results in G2 arrest and elongation of the cells, and subsequent release into inhibitor-free medium allows for synchronous re-entry into the cell cycle [13, 37]. However, as for a broad range of small hydrophobic molecules, the 3-MBPP1 inhibitor is absorbed by many of the most common materials used for microfabrication [19, 38, 39]. We therefore began by investigating the absorptive properties of the elastomer within the context of the microfluidic chips. To this end, we took advantage of the dose-dependent effects of 3-MBPP1 on analogue-sensitive fission yeast cells [13, 37]. Indeed, while Rhodamine B absorption is generally used to characterize this aspect of microfabrication materials [26, 40–42], our previous studies revealed that, in contrast to our cell-based assay, this method is not sufficiently sensitive to determine the compatibility of a given material with the use of small hydrophobic molecules [13]. Analogue-sensitive cells were injected in an elastomer chip coated with lectin and subsequently exposed to a constant flow of medium (20 μL/min) containing 0.4 or 1 μM 3-MBPP1 at 32 °C. When cells are grown in culture flasks, these concentrations of inhibitor induce a complete G2 arrest [37]. After 3 h (a cell cycle is 2 h 40 min in these conditions), we monitored the percentages of dividing cells in a population using septation as a marker. Our results showed a significant absorption at the border of the channels, where cells are in close proximity with the elastomer, resulting in high septation indexes (Fig. 5a, b, Additional file 3). Importantly, when focusing on cells further away from the material, the impact of this absorption was reduced, and cells at about 1 mm from the borders of the channels were fully arrested, similarly to the controls (Fig. 5a, b). These results demonstrate that when using small hydrophobic molecules in the elastomer chips, particular attention must be paid to discard results obtained from cells in close proximity to the material.

**Fig. 6:**
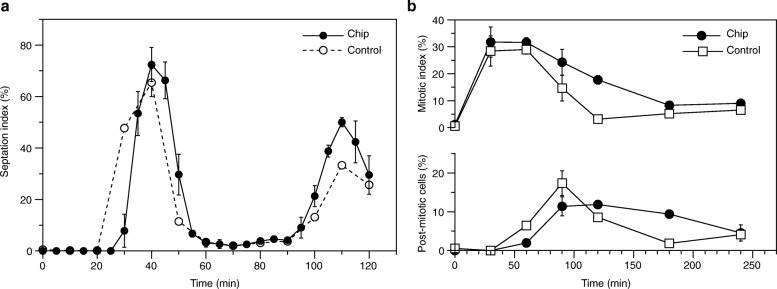
Monitoring cellular responses to dynamic changes in their environment. **a**. Analogue-sensitive fission yeast cells [37] were injected in a lectin-coated chip (as in Additional file 4C) and perfused with 1 μM 3-MBPP1 (20 μL/min) at 32 °C for 2 h 45 min (G2 block) before being released in inhibitor-free medium (T = 0, 20 μL/min). Control: cells grown in batch cultures at 32 °C, treated with 1 μM 3-MBPP1 for 2 h 45 min and released into inhibitor-free medium by filtration (T = 0). The septation indexes were then monitored over time (n > 50 for each time point). Averages of 3 independent experiments are shown with standard errors. Note that images were acquired every 5 min for the chips (mounted on a microscope) but every 10 min for the flasks, for which 5 min intervals were difficult to achieve. This led to a higher variability in timing for the chip assays compared to the less resolved batch cultures. **b.** HeLa cells were injected in a microsystem (as in Fig. 4c) maintained at 37 °C on a high precision hot plate to facilitate this proof-of-concept assay. Cells were then allowed to adhere for 3 h and subsequently treated with 6 μM of the CDK inhibitor RO-3306 for 10 min (13 μL/min) every hour for a total of 20 h. Cells were subsequently released in inhibitor-free medium (T = 0; 15 min at 20 μL/min and then 5 μL/min), and the percentages of mitotic (rounded) and post-mitotic (doublets) cells were quantified over time. Measurements were made in an area within 1.5 mm on each side of the center of the channel. As a control, cells treated with 6 μM RO-3306 in dishes were washed and incubated in normal medium. Averages of 3 independent experiments (*n* > 100 for each experiment) are shown with standard errors

Subsequently, we performed an inhibitor block and release assay in the chips using a variation of the approach described above. After exposing cells for 2 h 45 min to 1 μM 3-MBPP1 at 32 °C (one cell cycle in these conditions), we switched to inhibitor-free medium (20 μL/min) for an additional 2 h and assessed the septation index over time (Fig. 6a). As a control, the same experiment was conducted in batch cultures, inducing the release by filtration [37]. Our results showed a slight delay in cell cycle re-entry in the microsystems compared to the controls (~ 5 min), likely resulting from differences in the release kinetics. Indeed, while the medium switch is virtually instantaneous when using filtration, the time required to change the medium in the chips at this flow rate is consistent with this 5 min delay (Fig. 3b). Importantly, we systematically observed a better synchrony for cells in the chips, with the septation index reaching higher maxima for two successive cell cycles after the release. We hypothesize that this improved synchrony is the result of a more controlled and stable overall environment in the chips compared to the filtration protocol, which imposes more stress on the cells.

Finally, we performed a similar synchronization experiment using HeLa cells and the CDK1 inhibitor RO-3306 [43]. Cells resuspended after trypsin treatment were injected in the chips or in standard culture dishes and allowed to re-adhere to the surfaces for 3 h at 37 °C. 6 μM RO-3306 was then added to the control cultures or perfused to the cells in the microdevices for 20 h. In the latter case, the flow was triggered for 10 min every hour at a flow rate of 13 μL/min. This periodicity was implemented as a constant flow of fresh inhibitor over such a long period of time was toxic to the cells (our unpublished observations); this effect is not seen in the control cultures, in which the inhibitor is not constantly renewed. Cells were then released in inhibitor-free medium for 15 min at 20 μL/min and then maintained at a constant flow rate of 5 μL/min. Controls were subjected to a single medium switch. The percentages of both mitotic cells (rounded) and post-mitotic cells (duplets) were then determined. We observed similar profiles in both the chips and the controls, however with somewhat different fine kinetics (Fig. 6b). This is likely due to the major differences in culture conditions between the two approaches. In addition, cells may still be affected by some toxicity of the RO-3306, despite our protocol of periodic rather than constant renewal. Nevertheless, together with our biocompatibility assays in the presence of a constant flow (Fig. 4c), this demonstrates that media switches and the analyses of the responses of cells to such treatments in real-time are also possible for mammalian cells in our microsystems.

## Discussion

Microfluidic technologies represent a powerful approach for investigating cell biology, in particular when coupled to high-resolution fluorescence microscopy. However, they are not used to their full potential in research laboratories due to the complexity of the procedures for microdevice fabrication as well as to the requirement for expensive equipment and specific expertise. We have developed a system that is easy to fabricate and use, taking advantage of cost-effective solutions in its simplest version while still providing a number of advantages associated with microfluidics. These chips are re-usable, biocompatible, easily coupled with high-end photonic microscopy and allow for a dynamic control of the cellular environment during live-cell imaging assays. Furthermore, in contrast to the commercially available systems, they remain customizable and permit the implementation of a range of fluidic networks, including the multiplexing of several experiments on a single chip.

While simple to implement, this methodology has certain limitations. Specifically, it does not allow the fabrication of microsystems with the complexity and resolution obtained when using high-end clean room equipment. For instance, multi-layered chips that require advanced photolithographic processes cannot be constructed using our method. In addition, as we found that the LSR material absorbs small hydrophobic molecules, a well-known issue when using PDMS, our devices are especially suited when such treatments are not necessary. However, as discussed above, experiments involving small molecules are possible, but particular attention must then be paid to testing the interference of the LSR with each compound before use and adapting the experimental conditions accordingly. In addition, the scoring area must be restricted in order to limit the influence of the material’s absorptive properties.

Nevertheless, while these microsystems do not cover the full spectrum of microfluidic-based experiments for cell biology, they remain well-adapted to a broad range of assays that require the real-time monitoring of living cells in optimal and highly controlled growing environments. Furthermore, the thickness of the material makes these devices suited for the use of more complex multicellular systems, from organoids to embryos. Together with the simplicity, limited associated costs and compatibility of the described chips with live-cell imaging approaches, these features are likely to promote the expansion of microfluidics to laboratories that do not have expertise in the development of microdevices.

## Conclusions

Microfluidic systems provide an unprecedented level of dynamic control over the cellular environment in a variety of experimental contexts, in particular for challenging live-cell imaging approaches. However, their complexity of fabrication and usage has been an obstacle to the integration of these technologies in a majority of research laboratories. Through the identification of a novel material, we propose a simple approach for the implementation of basic yet powerful microfluidic chips that can be achieved without extensive technical expertise and microfabrication equipment. These systems will allow a larger community of researchers to benefit from the use of microfluidics for cell biology.

## Methods

### Fission yeast strains and methods

Standard media and methods were used [44, 45]. The strain used in this study was DC450 (*leu1Δ:Pcdc13::cdc13-L-cdc2as::cdc13–3’UTR::ura4*^*+*^ *cdc2Δ::kanMX6 cdc13Δ::natMX6 cig1Δ::hphMX6 cig2Δ::kanMX6 puc1Δ::leu2*^*+*^ *ura4-D18 h*^*+*^) [13]. The inhibitor-sensitive Cdc13-L-Cdc2as fusion protein was previously described [37]. All experiments were carried out in minimal medium plus supplements (EMM6S) at 32 °C. The 3-MBPP1 inhibitor (A603003, Toronto Research Chemical Inc.) was dissolved in DMSO at a stock concentration of 10 mM and added to the medium at the indicated concentrations. Size at division was determined from DIC images using Fiji (National Institutes of Health). Generation time of cells grown in the chips was determined by live-cell imaging as the time between two successive septation events.

### Mammalian cell lines and methods

All experiments were performed using HeLa Kyoto cell lines stably expressing an H2B::mCherry fusion protein (from plasmid 21,045, Addgene; Gregory Eot-Houllier, unpublished data). Cells were grown in DMEM GlutaMax medium (Thermo Fisher Scientific) supplemented with 10% Fetal Calf Serum, 10 mg/L streptomycin (Life Technologies), 10 units/L penicillin (Life Technologies) and 25 mM HEPES (Sigma Aldrich). The CDK inhibitor RO-3306 (Selleckchem) was used at a final concentration of 6 μM. All control experiments were performed in 12-well polystyrene dishes. For injection in the microdevices, cells cultures in polystyrene dishes were washed with PBS, treated with trypsin and resuspended in culture medium.

### Microscopy

Imaging for the results presented in Figs. 2c-e, 3b-d, 4a-b, 5a-b and 6a was performed using an inverted Zeiss Axio Observer (Carl Zeiss Inc.) equipped with a Lumencor Spectra X illumination system and a Hamamatsu Orca Flash 4.0 V2 sCMOS camera. The data for Figs. 1f and 3c were obtained using a similar setup that is equipped with a spinning disc confocal head and a laser bench (Visitron GmbH). For both systems, images were acquired using Visiview (Visitron GmbH). The images displayed in Figs. 1c-e (bottom panel) and 4c were acquired using a Zeiss Axio Vert. A1 (Carl Zeiss Inc.) equipped with a Nikon 1 J3 camera and a PlasDIC system. All image analyses were performed using Fiji (National Institutes of Health).

### Microfabrication

#### Materials

6 mm-thick extruded Perspex® PMMA (poly methyl methacrylate) for the chip manifolds was purchased from Vink-France. The double-sided adhesive tape (142 μm thick) used for the temperature control layer is ARcare 90,880 (Adhesives Research Inc.). The 250 μm-thick elastomer used for fabricating all the perfusion channels is Liquid Silicone Rubber (LSR) HT6240 40SH from Silex Silicones Ltd. UK. Glass coverslips (170 μm thick) were purchased from Menzel-Gläser, and plastic coverslips (Rinzel 72,261–60, 280 μm thick) are from Electron Microscopy Sciences. Bonding of the plastic coverslips to the PMMA manifolds for Figs. 4c and 6b was achieved using epoxy adhesive (Sader).

#### Chip dimensions

The designs and dimensions of the different layers constituting the chips described in this study are presented in Additional file 4.

#### Fabrication of chips for short-term experiments

The PMMA manifolds, temperature control layers and perfusion channels were obtained in the appropriate materials using a CO_2_ laser cutter (Speedy 100, Trotec, Austria). All designs were made using Corel Draw. The cutting parameters (laser power and speed) must be optimized for the laser machine used. To prevent deterioration when exposed to solvents, PMMA blocks were cured for 2 h at 90 °C. To assemble the chips, the manifolds were first bonded to the temperature control layers (fabricated in double-sided adhesive) and then to glass coverslips containing connecting holes generated using the CO_2_ laser cutting machine. The elastomer perfusion channels were then manually mounted (slowly depositing the LSR sheet onto the manifold layer from one side to prevent the formation of bubbles), sealing the PMMA/adhesive top layers with microscopy-grade glass coverslips. Rigid Teflon tubing (Cluzeau) was used to connect the chips to the temperature control machine as well as to the flow control system; the connections were sealed using epoxy glue. For the perfusion layers, the inner diameters of the tubings were 0.5 mm (upstream) and 1 mm (downstream), thereby limiting the impact of the chip fluidic resistance on the fluidic networks.

#### Fabrication of chips for long-term experiments

For long-term experiments, the glass coverslips separating the temperature and perfusion layers were replaced by plastic coverslips (Electron Microscopy Sciences, Rinzel 72,261–60, 280 μm thick). This was due to the relative weakness of the glass coverslips after laser cutting of the connecting holes. In this case, the temperature control layers were directly engraved within the PMMA manifolds using the CO_2_ laser machine, and bonding to the plastic coverslips was achieved by stamping of epoxy glue. Connecting holes in the plastic coverslips were made using a drilling machine. The other layers of the chips were identical to those of the devices fabricated for short-term experiments. For simplicity, all the proof-of-concept experiments using mammalian cells were performed using similar chip structures but without the temperature control channels (direct bonding of the plastic coverslip to the manifold). The temperature of the samples was then maintained using high precision hot plates.

### Chip preparation and cell loading

All mounted chips were sterilized by treatment with UV for 15 min prior to use. For experiments with non-adherent fission yeast cells, the bottom glass coverslip was coated with lectin (Sigma Aldrich). This was achieved by injecting lectin (0.5 mg/mL) in the chips using manual syringes. After a 30 min incubation, chips were thoroughly rinsed with deionized water, and cells were manually injected in the system. For experiments with adherent mammalian cells (Fig. 6b), glass coverslips were similarly coated with fibronectin (0.5 mg/mL) and rinsed with culture medium prior to cell injection. Resuspended cells were then injected and allowed to re-adhere to the glass substrate in a wet chamber placed at 37 °C in a CO_2_ incubator. Chips were kept in these conditions for a minimum of 3 h prior to applying any flow of medium.

### Control of the cellular environment in the microfluidic chips

Control of the temperature within the chip was calibrated and performed as previously described [13, 46], taking advantage of fine temperature regulation by Peltier elements (Cherry Biotech). The precise dimensions of the temperature control channels for the calibration were slightly different than for the layers used in the chips (see Additional file 4): while the thickness was the same, the length and width were altered to facilitate the connection of the metal electrodes to the measurement device (model 34972A from Agilent Technologies). Importantly, using this microfluidic system, the temperature of the sample relies on the thickness of the perfusion channel as well as the flow rate in the temperature channel (≥5 mL/min according to manufacturer’s instruction, which was validated in all our chips). Thus, the calibration performed using the modified temperature channel is valid for all chips described in this study. Control of the medium flow rates was achieved using flow sensors that regulate pressure controllers (Elvesys), and a valve matrix permitted rapid media switches (Elvesys). To limit the presence of bubbles within the microfluidic networks, bubble traps (Elvesys) connected to vacuum pumps (KNF) were mounted upstream of the chips. See Fig. 3a for a schematic of the full system. The application of a constant flow or frequent renewal of medium is necessary to maintain optimal growing conditions. In this context, the environment of the cells is imposed by the medium injected rather than by exchanges through the LSR material.

### Cleaning of the chips for reusability

The chips developed in this study can be re-used several times, with the bottom glass coverslip being the only single-use component of the entire system. The LSR layer can easily be peeled off manually from both bottom and top layers. Prior to re-use, all chips were dismantled, and all tubings were thoroughly washed with deionized water, 70% ethanol and dried using pressurized air. The elastomer layer was cleaned in an ultrasound waterbath at room temperature for 15 min, subsequently washed with 70% ethanol and dried using pressurized air.

## Additional files

Additional file 1: Fabrication of the temperature control layer with a razor blade and biopsy puncher. The temperature control layer (as in Additional file 4C) can be fabricated in double-sided adhesive (see Methods) with a CO_2_ laser cutter (left) as well as by hand using a razor blade and biopsy puncher (right). (PDF 699 kb)

Additional file 2: Calibration of the temperature control system. A relationship between the temperatures of the thermalization fluid (T_i_, as imposed by Peltier elements), lens (T_lens_, as measured by a contact sensor) and sample (T_sample_, as measured by deposited metal electrodes on the calibration coverslip) was established (see Methods, chip as in Additional file 4B). With the temperature of the lens, this equation allows for the calculation of the temperature of the thermalization fluid that is necessary to reach the target sample temperature [13]. (PDF 112 kb)

Additional file 3: Small molecule absorption by the elastomer. **A.** Drop assay demonstrating the absorption of 3-MBPP1 by the LSR. 40 μL of a culture of analogue-sensitive fission yeast cells (see Fig. 5) treated with 1 μM 3-MBPP1 or DMSO were deposited on a glass coverslip or on a 250 μm-thick sheet of LSR and incubated at 32 °C for 3 h. While cells on glass were arrested in their cell cycle and elongated, cells on LSR continued to divide, demonstrating the absorption of the inhibitor by the elastomer. DIC pictures. Scale bars = 10 μm. **B.** Complete LSR chips were treated with medium containing DMSO or 10 μM 3-MBPP1 for 1 h 30 min to saturate the material (flow rate: 30 μL/min). The chips were then washed with culture medium for 30 min at the same flow rate. Cells were injected in the chips and maintained at 32 °C for 3 h without flow. We observed cell cycle arrest due to release of 3-MBPP1 that was absorbed by the material. This demonstrates the requirement for a constant medium flow when using small molecules that are absorbed by the material. DIC images. Scale bars = 10 μm. **C.** The position of the cells in the channel has no effect on their growth. Fission yeast cells were injected in a LSR chip and maintained at 32 °C under a constant flow (20 μL/min) of medium. Size at division was determined after 3 h at the border of the LSR or between 1.8 and 2 mm away from the edge of the channel (*n* ≥ 40 for each measurement). Standard deviations are shown. (PDF 1466 kb)

Additional file 4: Microfluidic designs used in this study. **A.** Schematic of the patterns in Fig. 1b. Dimensions are in mm. **B.** Schematics of the temperature and perfusion channels used for the calibration (Additional file 2) and for Fig. 2c-e. **C.** Schematics of the temperature and perfusion channels used for Figs. 2b, 3b-d, 4, 5 and 6. Diagrams are not to scale. (PDF 105 kb)
