# Supplementary figures and images for: An easy-to-build and re-usable microfluidic system for live-cell imaging
Source: BMC Cell Biol. 2018 Jun 20;19:8. doi: 10.1186/s12860-018-0158-z (PMC6011407; doi:10.1186/s12860-018-0158-z)

## Additional file 1

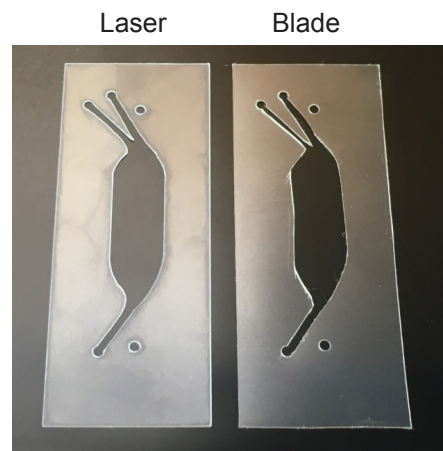

Supplement: Supplementary file 1 — Fabrication of the temperature control layer with a razor blade and biopsy puncher. The temperature control layer (as in Additional file 4C) can be fabricated in double-sided adhesive (see Methods) with a CO2 laser cutter (left) as well as by hand using a razor blade and biopsy puncher (right). (PDF 699 kb) [file 12860_2018_158_MOESM1_ESM.pdf]

## Additional file 2

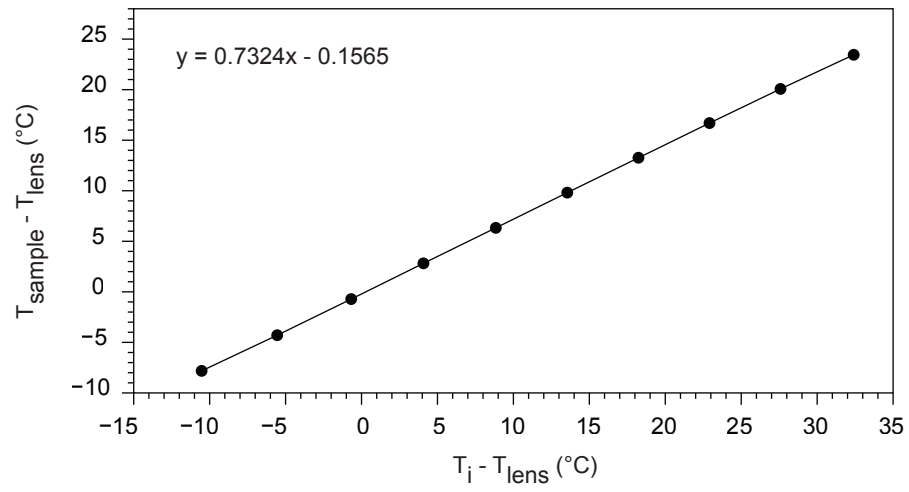

Supplement: Supplementary file 2 — Calibration of the temperature control system. A relationship between the temperatures of the thermalization fluid (Ti, as imposed by Peltier elements), lens (Tlens, as measured by a contact sensor) and sample (Tsample, as measured by deposited metal electrodes on the calibration coverslip) was established (see Methods, chip as in Additional file 4B). With the temperature of the lens, this equation allows for the calculation of the temperature of the thermalization fluid that is necessary to reach the target sample temperature [13]. (PDF 112 kb) [file 12860_2018_158_MOESM2_ESM.pdf]

Additional file 3

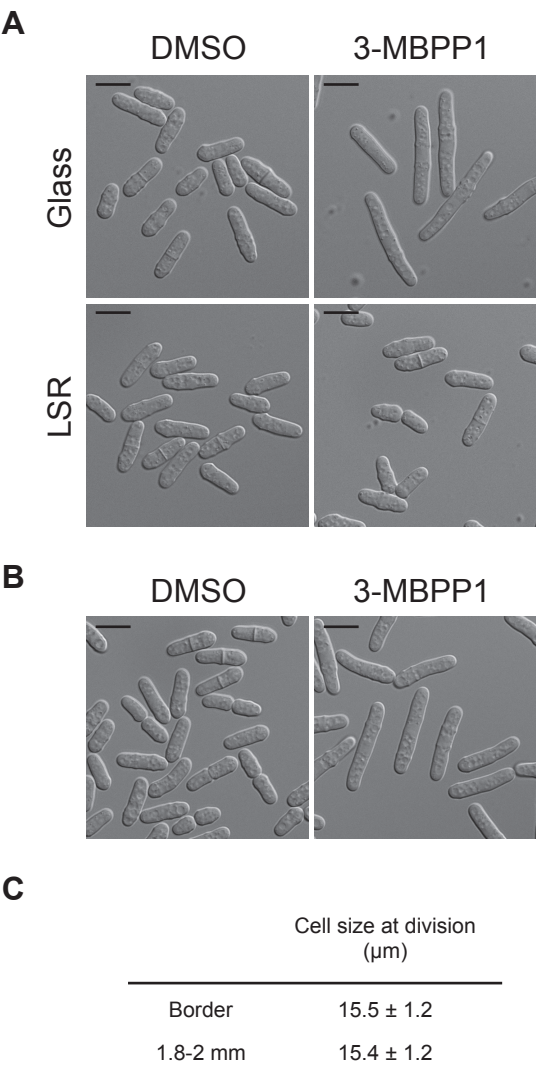

Supplement: Supplementary file 3 — Small molecule absorption by the elastomer. A. Drop assay demonstrating the absorption of 3-MBPP1 by the LSR. 40 μL of a culture of analogue-sensitive fission yeast cells (see Fig. 5) treated with 1 μM 3-MBPP1 or DMSO were deposited on a glass coverslip or on a 250 μm-thick sheet of LSR and incubated at 32 °C for 3 h. While cells on glass were arrested in their cell cycle and elongated, cells on LSR continued to divide, demonstrating the absorption of the inhibitor by the elastomer. DIC pictures. Scale bars = 10 μm. B. Complete LSR chips were treated with medium containing DMSO or 10 μM 3-MBPP1 for 1 h 30 min to saturate the material (flow rate: 30 μL/min). The chips were then washed with culture medium for 30 min at the same flow rate. Cells were injected in the chips and maintained at 32 °C for 3 h without flow. We observed cell cycle arrest due to release of 3-MBPP1 that was absorbed by the material. This demonstrates the requirement for a constant medium flow when using small molecules that are absorbed by the material. DIC images. Scale bars = 10 μm. C. The position of the cells in the channel has no effect on their growth. Fission yeast cells were injected in a LSR chip and maintained at 32 °C under a constant flow (20 μL/min) of medium. Size at division was determined after 3 h at the border of the LSR or between 1.8 and 2 mm away from the edge of the channel (n ≥ 40 for each measurement). Standard deviations are shown. (PDF 1466 kb) [file 12860_2018_158_MOESM3_ESM.pdf]

Additional file 4

A

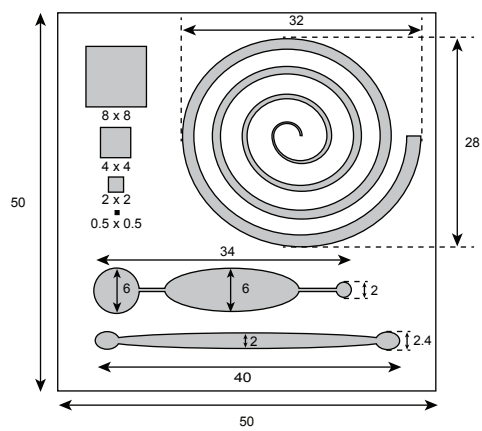

B

Calibration chips

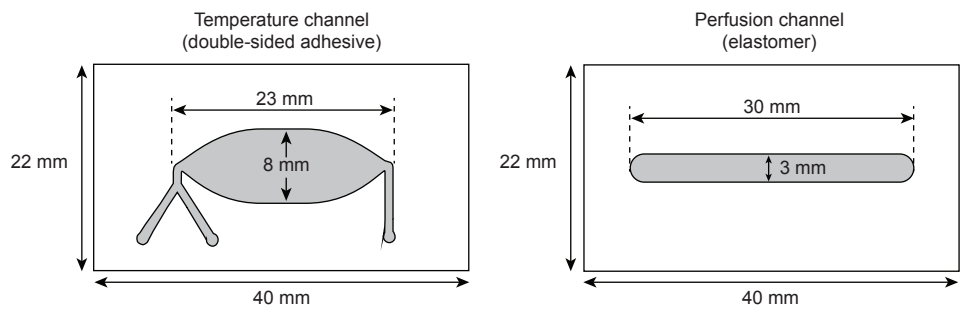

C

Experimental chips

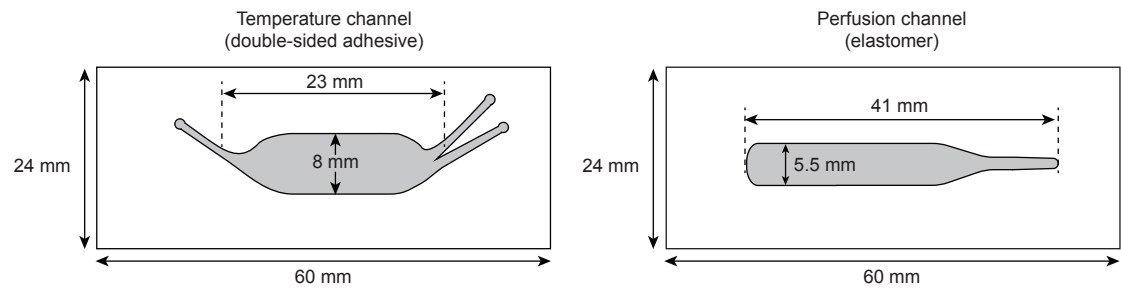

Supplement: Supplementary file 4 — Microfluidic designs used in this study. A. Schematic of the patterns in Fig. 1b. Dimensions are in mm. B. Schematics of the temperature and perfusion channels used for the calibration (Additional file 2) and for Fig. 2c-e. C. Schematics of the temperature and perfusion channels used for Figs. 2b, 3b-d, 4, 5 and 6. Diagrams are not to scale. (PDF 105 kb) [file 12860_2018_158_MOESM4_ESM.pdf]
